# Experimental validation of Davis LUT module with photonic crystals for GATE simulations

**DOI:** 10.1088/1361-6560/ae752d

**Published:** 2026-06-16

**Authors:** Xuzhi He, Nicolaus Kratochwil, Gerard Ariño-Estrada, Vinay Jain, Emilie Roncali

**Affiliations:** 1 Department of Biomedical Engineering, University of California at Davis, Davis, CA 95616, United States of America; 2 Department of Radiology, University of California at Davis, Sacramento, CA 95817, United States of America

**Keywords:** positron emission tomography, photonic crystals, detector, optics, inverse design

## Abstract

Positron emission tomography (PET) image quality is partially determined by PET detector performance, which can be improved by increasing the optical photon harvest from scintillator to photodetector. Due to the steep index mismatch at this interface, the optical photon critical angle limits the harvest. Photonic crystals (PhCs) are periodic nanostructures that can break through the critical angle limit, with a size comparable to the optical photon wavelength. Previous work including both simulation and experimental studies indicated that PhCs can enhance PET detectors’ optical photons harvest. However, computational models can be inaccurate due to the necessity of using wave optics modeling, which can be challenging when modeling fabricated PhCs due to physical defects (e.g. loss of periodicity). Our group has developed a new computational method based on the previous look-up-table (LUT) Davis model implemented in the Monte Carlo simulation software GATE. The novel method models fabricated PhCs, including defects: first, we used laser characterization to analyze non-ideal PhCs and then used a reverse design approach to find the actual geometry of these ‘Effective PhCs’ LUT. We built the ‘Effective PhCs’ LUT and incorporated it into GATE. The new model was tested with 3 × 3 × 20 mm^3^ lutetium yttrium oxyorthosilicate (LYSO). We compared the simulated light collection results (pulse height spectra) with experimental characterization of the LYSO-PhCs detector performance and showed the new model provided better prediction of the light collection than previous models, opening a path to model fabricated PhCs with imperfect geometries in Monte Carlo simulation tools such as GATE and Geant4. Although the light collection of LYSO-PhCs detectors was less than that of the LYSO-No PhCs detectors, the ‘Effective PhCs’ light prediction can advance the scintillator-PhCs detector design and guide the fabrication to reduce PhCs defects so that we can improve PET detector’s performance.

## 1. Introduction

Lutetium oxyorthosilicate and lutetium yttrium oxyorthosilicate (L(Y)SO) are the dominant commercial positron emission tomography (PET) detector scintillator materials (Moses 2011). One of the limitations in PET detector detection efficiency arises from total internal reflection at the scintillator-photodetector interface. Optical photons emitted from gamma ray-scintillator interactions impinging on the interface with incidence angles larger than the critical angle may be trapped inside the scintillator and eventually get absorbed by the scintillator or transmitted and lost through the scintillator’s side surface. This phenomenon reduces the PET detector’s light collection (LY_coll_) and is detrimental to energy resolution and ultimately to image quality. Photonic crystals (PhCs) are periodic nanostructures with size comparable to optical wavelengths that can break through the critical angle limit. Several groups have proposed various strategies to mitigate this limitation, such as advanced optical coupling materials (Lecoq *et al* 2013) (Lecoq *et al* 2007). These groups mainly used experimental methods to prove that scintillation detectors’ LY_coll_, energy resolution (*E*_r_) and timing resolution could be enhanced with the application of PhCs (Salomoni *et al* 2018, Knapitsch and Lecoq 2014, Pots *et al* 2019, Francesco *et al* 2021).

More recently, our group developed a method to incorporate a PhCs model into the Monte Carlo simulator GATE for scintillation detectors, based on the look-up-table (LUT) Davis model by following wave optics instead of geometry optics theory. We found that simulation results from PhCs models matched experimental results mentioned before reasonably well (Salomoni *et al* 2018, He *et al* 2025). These results established a simulation-driven pathway for the development of advanced PET detectors utilizing PhCs and requiring wave optics models, enabling cost-effective and efficient design optimization.

**Figure 1.**
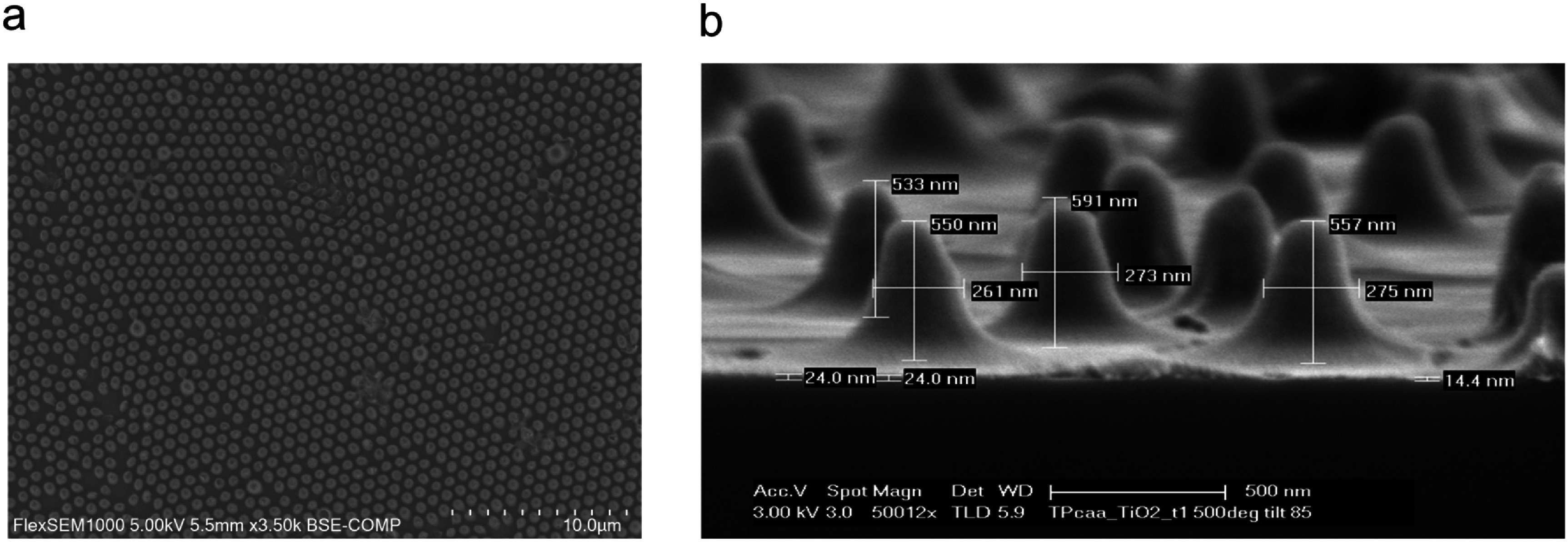
SEM imaging of photonic crystals’ top view (a) and side view (b). (a) The top view shows that the PhCs’ unit cells follow an hexagonal lattice pattern. However, some empty regions may exist between unit cells. Some unit cells’ shapes are distorted. (b) Most unit cells can be approximated as a Gaussian shape but with varying height.

Nevertheless, fabricated PhCs often exhibit defects (figure 1(a)) that prevent accurate modeling of their performance with a finite difference time domain (FDTD) framework, needed for these periodic nanostructures. Here, we propose a novel method to model the performance of PhCs with defects, denoted by ‘non-ideal PhCs’. With the aim of overcoming these challenges, we combined experimental and simulation methods to reverse-engineer the effective PhCs nanostructures and input it into Ansys Lumerical FDTD. That way we were able to build ‘Effective PhCs’ optical LUT for GATE simulations (Roncali and Cherry 2013, Stockhoff *et al* 2017, He et al 2025). In this work, we first experimentally characterized the LYSO–PhCs interface by illuminating it with a laser and measuring the corresponding far-field distribution. We then systematically varied the Gaussian-profile unit cell height and lattice distance in Ansys Lumerical FDTD simulations and computed the associated far-field projections. By comparing the simulated far field patterns with the measured ones, we identified the unit cell geometry that yielded the closest agreement. This optimized nanostructure is hereafter referred to as ‘*Effective PhCs’* unit cell. The vendor (NAPA Technologies, Haute Savoie, France) provided us with LYSO-PhCs samples (as illustrated in figure 1) for modeling and characterization. This sample has a size of 3 × 3 × 20 mm^3^. In our scintillation detector characterization experiment, the LYSO was wrapped with Teflon on the five polished faces without PhCs and a Broadcom silicon photomultiplier (SiPM) was coupled with the LYSO-PhCs interface with a glue of refractive index 1.47 to match the PhCs’ encapsulation material silicon dioxide (SiO_2_). By comparing the light yield results, we could demonstrate the superior accuracy of the new ‘Effective PhCs’ model over the model in He *et al* (2025).

## 2. Materials and method

### 2.1. Scintillator and PhCs materials and fabrication

**Figure 2.**
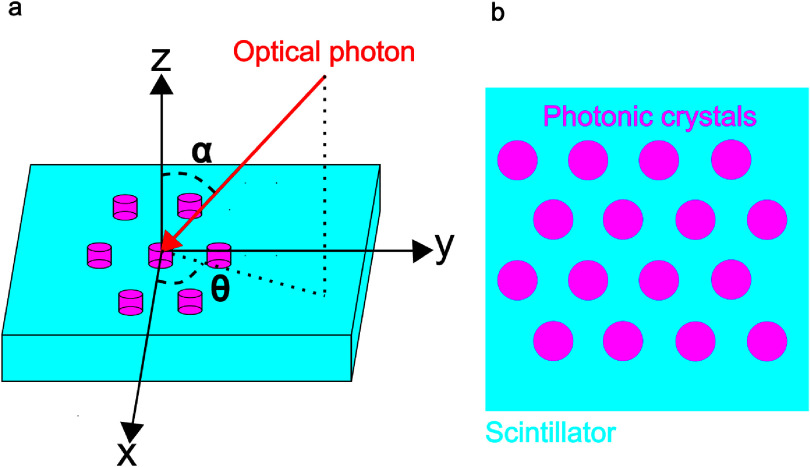
Procedure to produce photonic crystals LUT. (a) Optical photons illuminate the scintillator-PhCs interface (illustrated by the red arrow). *α* is the incident polar angle and *θ* is the azimuth angle. The cyan block represents the scintillator, and the magenta cylinders represent the PhCs material. Both angles are varied to simulate various angles of incidence (*α*) and orientations (*θ*). (b) Top zoom-in view of scintillator-PhCs.

Before summarizing our modeling strategy, two terms need to be defined: (1) the far field is the energy distribution resulting from the transmission of a laser shone upon PhCs. (2) Lattice distance refers to the distance between PhCs’ unit cells (figures 1(a) and 2(b)). PhCs fabrication defects: imperfect periodicity of the unit cells pattern, presence of bump regions between unit cells, geometry variation between unit cells and from the nominal geometry (figure 1). For example, the nano-cylinder described in Salomoni *et al* (2018) may not be rendered as a perfect cylinder and may exhibit a Gaussian profile instead due to fabrication process limitations. This limits our ability to accurately set up the unit cell structure in FDTD PhCs simulation conducted in Ansys Lumerical. Figure 1 shows the SEM imaging of the PhCs’ top and side views. These SEM images were obtained from the vendor during the fabrication, carried out with nanoimprint technology to deposit titanium dioxide (TiO_2_, used for PhCs material) onto the LYSO scintillator surface. As shown in figure 1(a), the PhCs pattern approximately follows the hexagonal lattice pattern with average lattice distance of 632 nm with standard deviation of 11 nm. Figure 1(b) depicts the PhCs’ unit cell geometry, revealing more a Gaussian shape than a conical geometry.

### 2.2. Detector modeling with LUT Davis model

To build the PhCs LUT, we followed the computational procedure described in He *et al* (2025). This procedure, done in Ansys Lumerical, can be summarized as: (1) we illuminated the scintillator-PhCs interface with an optical wave (red arrow in figure 2(a)) (2) we varied the illumination polar angle *α*, azimuth angle *θ* and wavelength. (3) We averaged the transmission coefficients and projected the energy distribution which is ultimately used to build LUT. Figure 2(b) shows the top zoom-in view of PhCs pattern. In this schematic (figure 2(b)), the scintillator is shown in cyan, and the PhCs structures are represented by the magenta disks. Finally, we incorporated the PhCs LUT into GATE to simulate LYSO-PhCs detector light collection. In GATE, we set all 5 remaining surfaces as polished and wrapped with Teflon.

**Figure 3.**
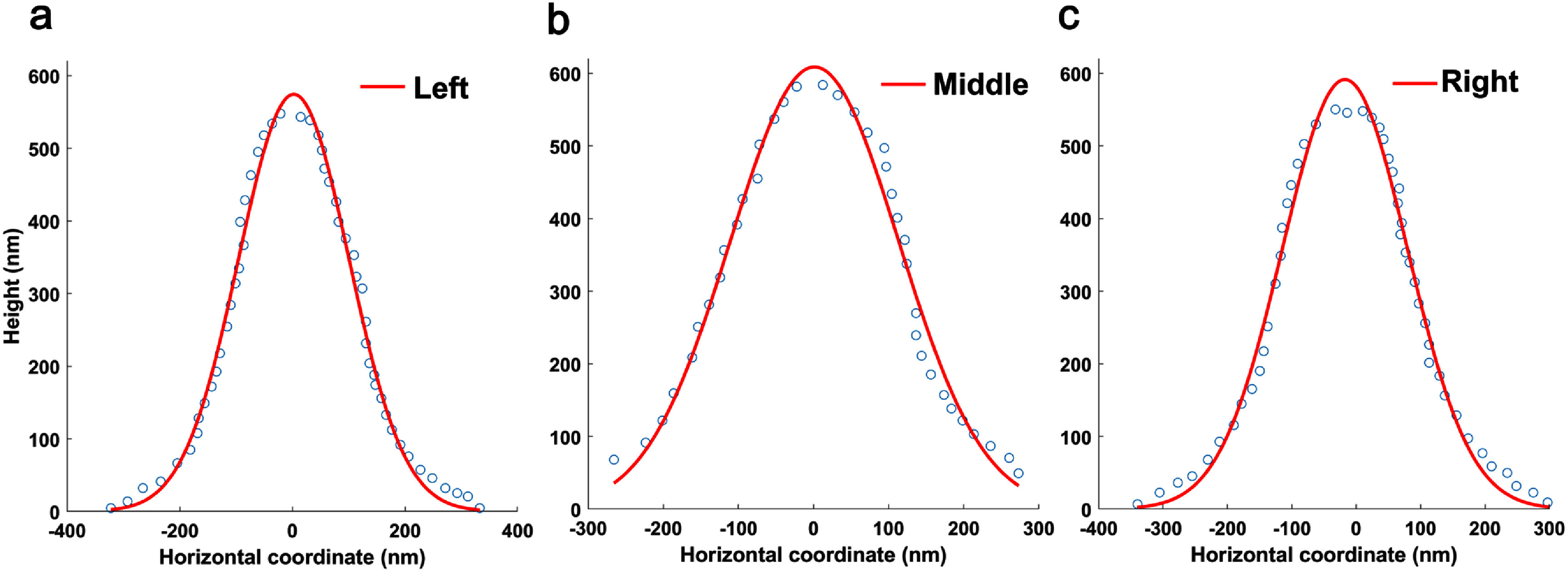
Extracted unit cell profile data from figure 1(b). Legend ‘Left’, ‘Middle’ and ‘Right’ represent three geometries of unit cells’ side view in figure 1(b)

We extracted the three-dimensional coordinate data of the unit cells’ profiles from figure 1(b) and presented illustrative profiles in figure 3. We then used a Gaussian model to fit the three profiles from figure 3 and averaged the fitted Gaussian parameters, and obtained the following analytical Gaussian profile equation:

\begin{align*}{\mathbf{Height}} = {\boldsymbol{591.42}}{\boldsymbol{*}}{{\mathbf{e}}^{ - {\text{ }}\frac{{{{\mathbf{x}}^{\boldsymbol{2}}} + {{\mathbf{y}}^{\boldsymbol{2}}}}}{{{{{\boldsymbol{144.7}}}^{\boldsymbol{2}}}}}}}\end{align*}

where *x* and *y* represent the horizontal coordinates on the scintillator surface.

**Figure 4.**
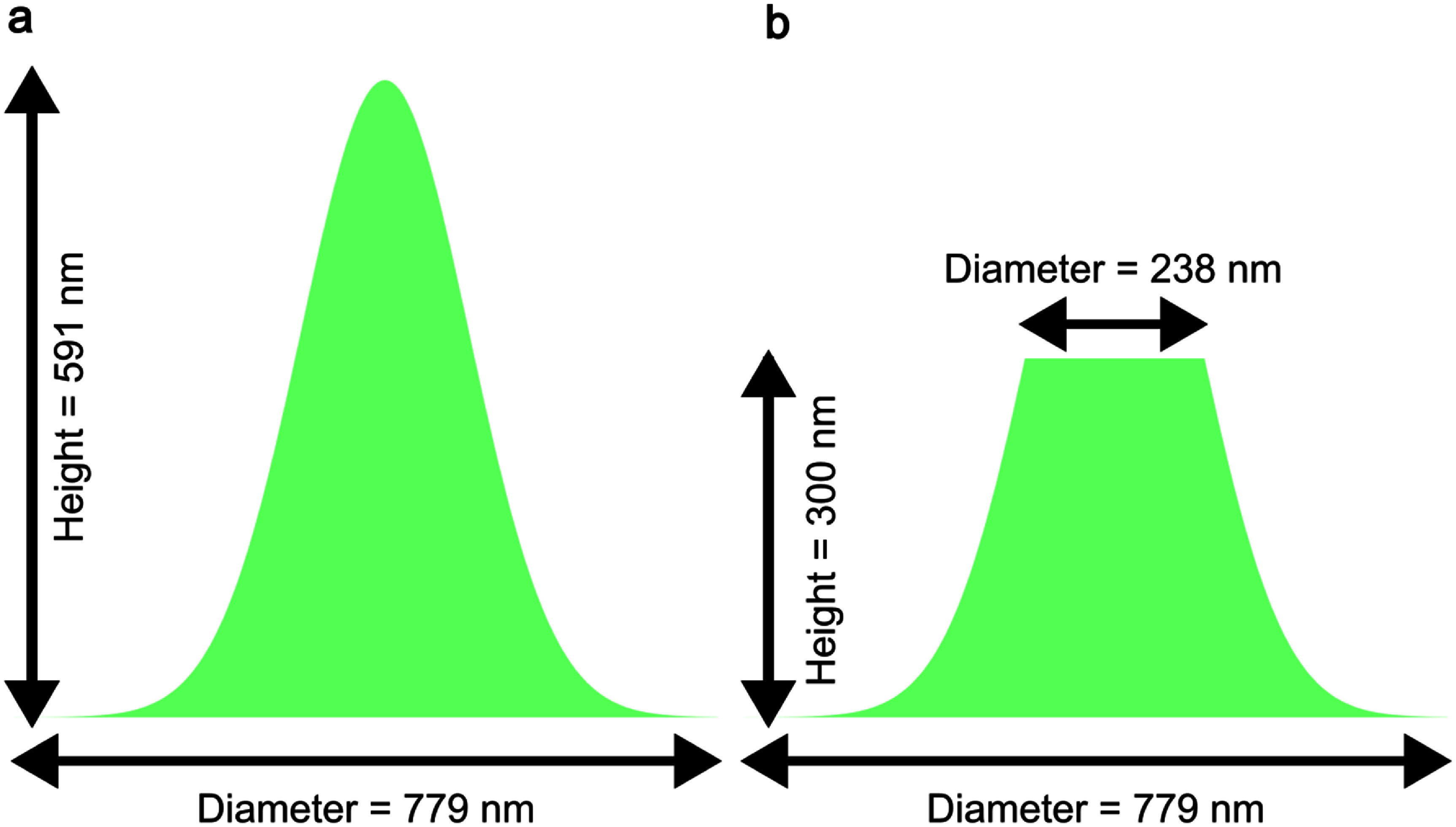
Cross-section geometry of PhCs unit cell used for nanophotonic simulation. (a) The SEM PhCs unit cell was modeled as a perfect Gaussian profile. (b) Compared to the SEM profiles, the effective PhCs unit cell is modeled with a lower height of 300 nm.

We incorporated the Gaussian 3D structure based on equation (1) (also shown in figure 4(a)) with a height of 591 nm and a diameter of 779 nm into Ansys Lumerical FDTD and set the lattice distance to 632 nm. In the FDTD simulation, we considered three incident azimuth angles *θ* of 0°, 30° and 60° corresponding to the PhCs hexagonal lattice pattern shown in figure 1(a); 91 polar angles *α* ranging from 0° to 89.9° with a step size of 1°, 30 wavelengths ranging from 300 nm to 800 nm with a step size of 17.24 nm. All angles are defined in figure 2(a). The boundary condition of unit cell was defined as a periodic boundary. The PhCs LUT was built after simulating the PhCs with the three parameters above (wave incident azimuth angle, wave incident polar angle and wavelength). This LUT is denoted by SEM PhCs LUT and this PhCs structure is denoted by SEM PhCs. The next section details an alternative approach to building the PhCs LUTs based on experimental optical characterization.

### 2.3. Reverse design of laser characterized PhCs process

#### 2.3.1. Effective PhCs definition

Several types of defects were visible on fabricated PhCs that make them non-ideal periodic structures, as shown in figure 1(a). These defects made it difficult to accurately model the PhCs’ far field based on lattice distance extracted from SEM images only. In addition, SEM images can only be collected during limited fabricated steps, and post-encapsulation characterization is not feasible. We developed a new method to model these non-ideal PhCs by combining experiments with simulations. We first shot a laser upon a scintillator-PhCs interface and measured the experimental far field. Secondly, we varied the PhCs’ unit cell geometry (lattice distance) and projected far field parameters in Ansys Lumerical FDTD. Finally, we chose the parameters providing the closest match between simulated projected far field and experimental far field. These parameters were used to update the PhCs unit cell geometry and simulation parameters. This configuration is denoted by ‘Effective PhCs’ and its corresponding LUT is denoted by ‘Effective PhCs LUT’ in the rest of the article.

#### 2.3.2. Far field characterization

**Figure 5.**
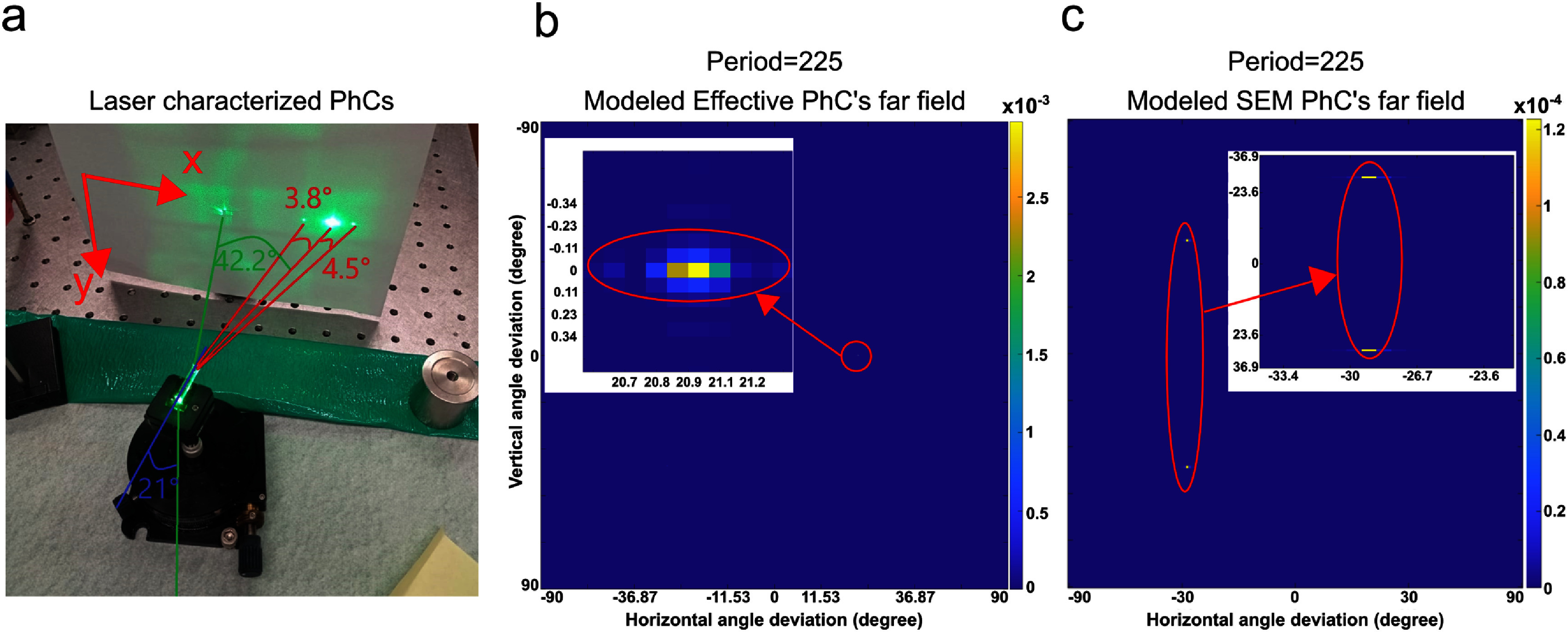
Process of reverse design PhCs. (a) Green laser used to characterize scintillator-PhCs interface on optical table to obtain far field, with a wavelength of 532 nm. (b) Simulated far field for the effective PhCs. (c) Simulated far field for SEM PhCs. Two hot spots are vertically separated, and this energy distribution is totally different from experimental results in (a), so (c) PhCs structures should not be input into Ansys Lumerical FDTD.

**Figure 6.**
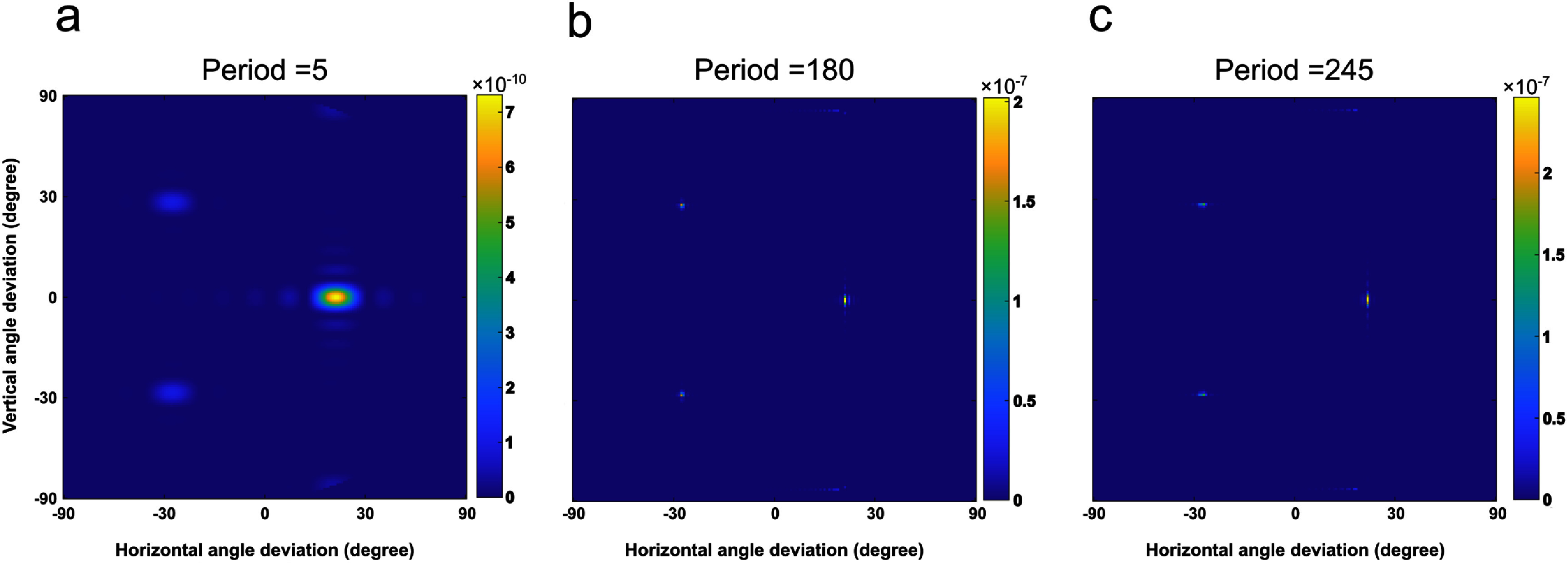
Simulated far field of ‘Effective PhCs’ geometry for three different period numbers. A top hat illumination was applied to all simulations. None of far fields matched the experimental far field shown in figure 5(a).

As shown in figure 5(a), we directed a laser beam with a wavelength of 532 nm onto a 3 × 3 × 20 mm^3^ LYSO crystal. The scintillator was placed on a rotation stage. The laser impinged on the scintillator’s entrance surface (polished) with an incidence angle of 21° between the green line and blue line (LYSO entrance surface’s normal vector). The resulting far field was imaged on a screen and contained three hot spots. Some scatter spots, indicated by the green line between scintillator’s exit surface and white board, relatively parallel to incident laser beam, are from the scintillator’s side surface. The red lines indicate the PhCs’ far field. The angle subtended by the central red line and the upper green line represent the central hot spot angle deviation, around 21°, which is consistent with the angle deviation in figure 5(b). The other two hot spots with horizontal deviation (3.8° and 4.5°) geometrically match with two horizontal hot spots’ angle deviation in figure 5(b) reasonably well. Considering the unit cell geometries in figure 1(b) were scanned before using SiO_2_ to encapsulate PhCs, which may have altered the geometry (shrinking height), we varied the lattice distance from 600 nm to 800 nm with step size of 10 nm to find the closest match between the measured and simulated far fields. Two parameters played a pivotal role when finding the Effective PhCs’ structure with FDTD: (1) the PhCs periodic number and (2) the illumination method. The ‘PhCs periodic number’ refers to number of PhCs’ columns, needed to project the far field from the computed near field. The far field comes from the coherent superposition of the near field radiated by each unit cell, so the number of unit cells affects the far field pattern. The ‘period number’ is used to project the far field from the near field. The ‘periodic boundary’ condition is used to determine the near field. In the computation, we varied the periodic number from 5 to 245 with step size of 5 and found the optimized periodic number that matched the experimental pattern was 225. Far fields simulated for three different period numbers are displayed in figure 6 and illustrate the discrepancy between fields with other period numbers and the experimental far field in figure 5(a).

We varied the periodic number parameter because when the periodic number was larger than 245, the hot spot would become too small to match experimental results. There are two illumination methods in Ansys Lumerical FDTD: (1) Gaussian illumination in which the optical structure is much larger than the laser spot size, (2) Top hat illumination where the optical device is smaller than the laser spot size. Figure 5(a) shows the entry surface where the laser beam first hits the scintillator being uniformly illuminated, which indicates that the laser spot size was conspicuously larger than the scintillator’s cross-section of 3 × 3 mm^2^. Therefore, we selected the ‘Top hat’ illumination method. Finally, the ‘Effective PhCs’ structure had a 3D Gaussian shape with diameter of 779 nm, height of 300 nm, flat top, and lattice distance of 650 nm; meanwhile, the far field projection parameters were: periodic number of 225, top hat illumination so that the simulated far field can match the experimental far field. We incorporated the Effective PhCs (figure 4(b)) unit cell structure into Ansys Lumerical FDTD. Using these simulation parameters, we computed the far field of ‘Effective PhCs’ (figure 5(b)) which was in good agreement with the laser-characterized far field (figure 5(a)). There are three horizontal hot spots in figure 5(b) with similar appearance as the horizontal three hot spots in figure 5(a). The deviation angles have a small difference between (a) and (b) because LED light in experiments is incoherent while we shot a plane wave in Lumerical which represents the coherent light. However, Lumerical light source setting currently does not have incoherent light source option and can only approximate the coherent light source as incoherent light source. In contrast, we simulated the SEM PhCs’ far field by adopting the same far field project parameters (periodic number of 225, top hat illumination). Results shown in figure 5(c) have significant discrepancies against the laser-characterized far field (figure 5(a)). There are no two vertical $ \pm {30^{\mathrm{o}}}$ hot spots in the experimental far field, shown as figure 5(a), because ‘Effective PhCs’ (lattice distance of 650 nm and period number of 225) can represent the performance of ‘real fabricated PhCs’ structure. The period number was chosen to be 225 to best match the experimental far field. There is no link between the two vertical hotspots and height parameter of PhCs. There are three horizontal hot spots in figure 5(b) with similar appearances as the horizontal three hot spots in figure 5(a). In figure 5(a), the power ratio between the three hot spots in ‘Real PhCs’ far field is 0.35:1:0.47. In figure 5(b), the power ratio between the three hot spots in ‘Effective PhCs’ far field is 0.47:1:0.57.

**Figure 7.**
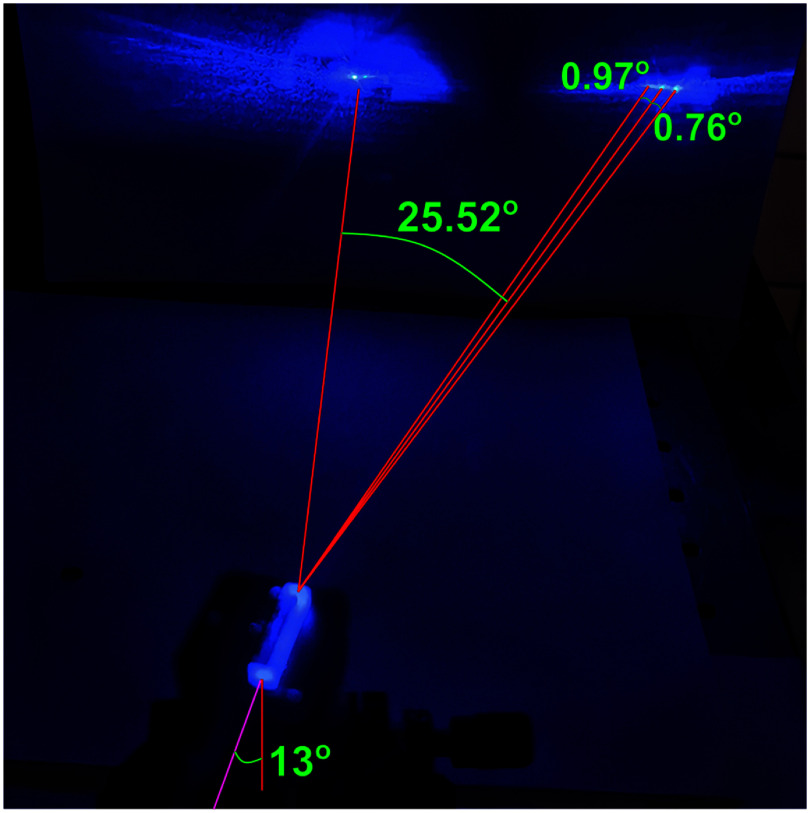
Scintillator-PhCs characterized at 440 nm.

We characterized the LYSO-PhCs performance by laser with another wavelength which is 440 nm, as shown in figure 7. This laser had lower power than the laser with green light in figure 5(a), the experiment was conducted in a dark environment. The laser incident angle at LYSO entrance surface was 13°. Few scattered light from LYSO side surface were relatively parallel to the incident ray. Three horizontal hot spots had deviation angles of 24.55°, 25.52°, and 26.28°. The power ratio between the three hot spots from left to right is 1:4.9:9.8 which was different from the power ratio in figure 5(a).

### 2.4. GATE detector simulations

All detector simulations using the different PhCs LUTs described above were carried out in GATE (Stockhoff *et al* 2017). A monoenergetic gamma point source with an energy of 511 keV and an activity of 10 000 Becquerels was used to irradiate a single crystal. The scintillation crystal was a 3 × 3 × 20 mm^3^ LYSO coupled with a SiPM. The solid angle of the gamma source emission was constrained to fully cover the scintillator cross section, with photons entering from the surface opposite to the photodetector. Four Davis LUTs were incorporated into scintillator-photodetector interface: (1) polished LYSO-Teflon (2) polished LYSO-glue (3) polished LYSO-SEM PhCs (4) polished LYSO-Effective PhCs.

### 2.5. PET detector experimental setup

The LYSO:Ce,Ca samples (3x3x20 mm^3^ were coupled to NUV-MT SiPMs from Broadcom (AFBR-S4N44P01M, 3.7 × 3.7 mm^3^ active area) and wrapped in multiple layers of Teflon. Each SiPM was biased at 40 V, which corresponds to an overvoltage of 7.5 V. The SiPM signal was amplified with custom high frequency readout electronics (based on the design of (Cates *et al* 2018), (Gundacker *et al* 2020)) and the signal was digitized with a Tektronix oscilloscope (MSO 64B, 4 GHz, 20 Gs s^−1^). The acquired waveforms were offline analyzed to extract the pulse height spectra under irradiation from a 22Na radioactive source, providing 511 keV and 1.27 MeV gamma emissions. No SiPM saturation correction was performed. In addition, we extract the crossing time for different leading-edge thresholds after baseline correction (Kratochwil *et al* 2025) and linear interpolation between the points. Coincidence time resolution was measured against a reference detector (small LYSO:Ce,Ca crystal) with a CTR (for two identical reference detectors) of 70 ps full width at half maximum (FWHM).

## 3. Results

### 3.1. LUT angular distribution of different optical coupling

**Figure 8.**
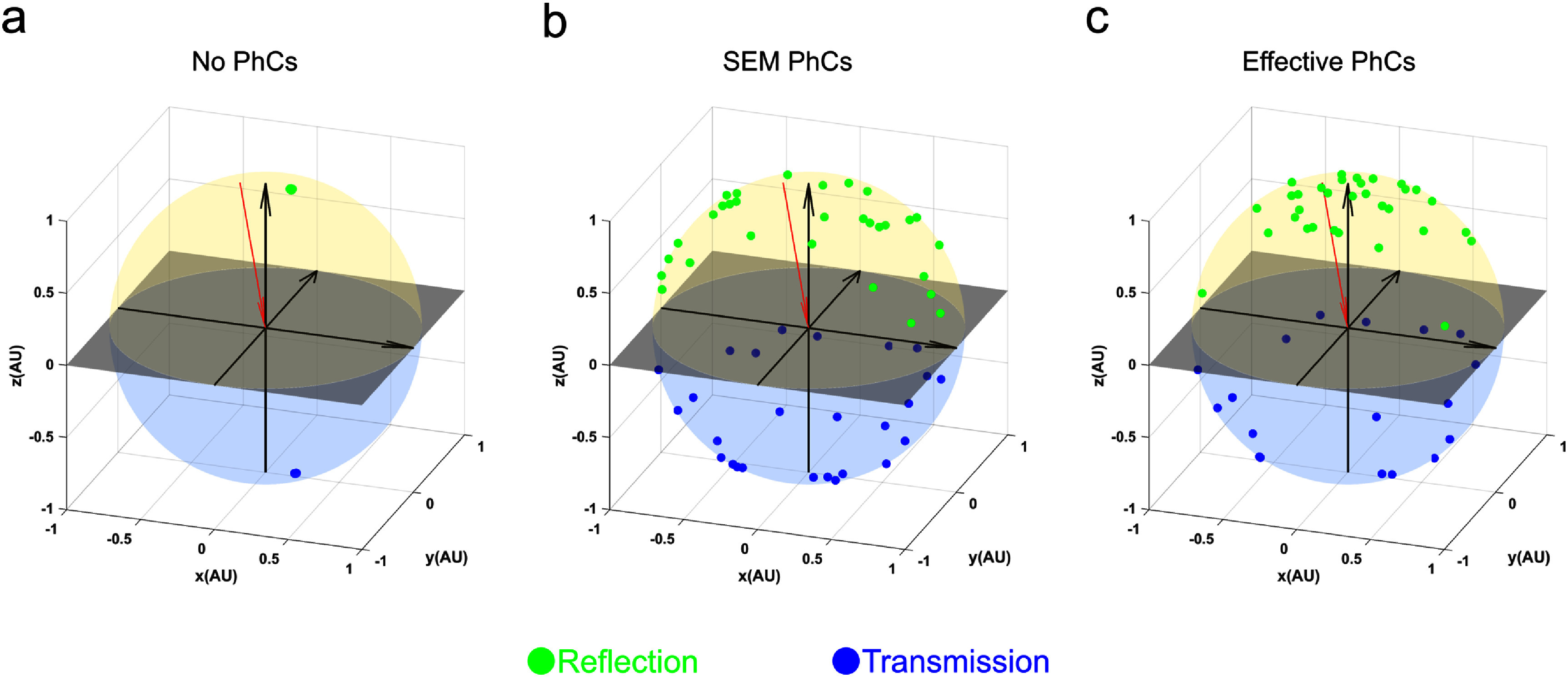
Optical photon angular distribution for different scintillator-photodetector coupling for an illustrative incident angle of 10° (shown in red). Red arrows represent incident optical photons direction. (Left) ‘No PhCs’ coupling. (Middle) ‘SEM PhCs’ coupling. (Right) ‘Effective PhCs’ coupling. The ‘No PhCs’ angular distribution is similar to optical specular interactions. Reflected and transmitted optical photons in ‘SEM PhCs’ spread out more uniformly. Reflected optical photons in ‘Effective PhCs’ tend to converge to the top sphere region while transmitted optical photons are more uniformly distributed.

Figure 8 shows the optical photon angular distribution for three different LUT when the incident optical photon angle is 10°. The red arrow represents the incident optical photon direction. Green dots and blue dots are reflected and transmitted optical photons, respectively. If an optical photon is incident upon the gray surface with angle of 10°, the optical photons may be reflected towards one of the green dots’ positions or transmitted towards one the blue dots’ positions. The left subplot is ‘No PhCs’ coupling which can be approximated as specular reflection. The ‘SEM PhCs’ and ‘Effective PhCs’ are shown in the middle and right subplots. These three angular distributions determine the collected optical photon angular distribution, LY_coll_ and eventually have an effect on PET detector performance.

### 3.2. Simulated transmissivity, pulse height spectrum and detection time distribution

**Figure 9.**
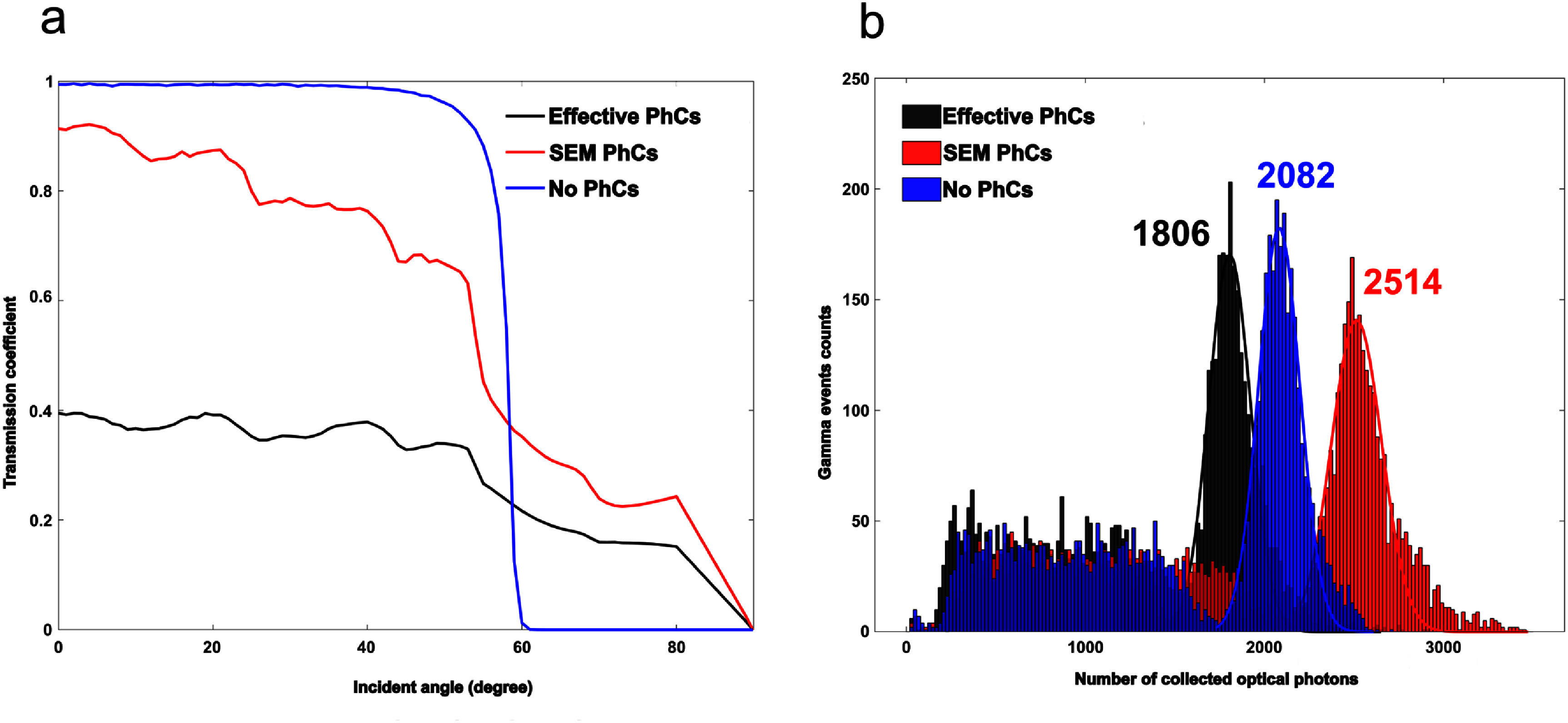
Simulation results. (a) Optical photons transmission curve for no PhCs, SEM PhCs, and effective PhCs. The critical angle and total internal reflection range is clearly marked for the polished interface without PhCs (blue curve) but strongly attenuated for the PhCs (red and black curves). These transmissivities were computed by Ansys Lumerical FDTD. (b) Pulse height spectra for the three PhCs LUTs showing a decreasing number of collected photons (based on the peak position) for the two PhCs LUTs.

**Figure 10.**
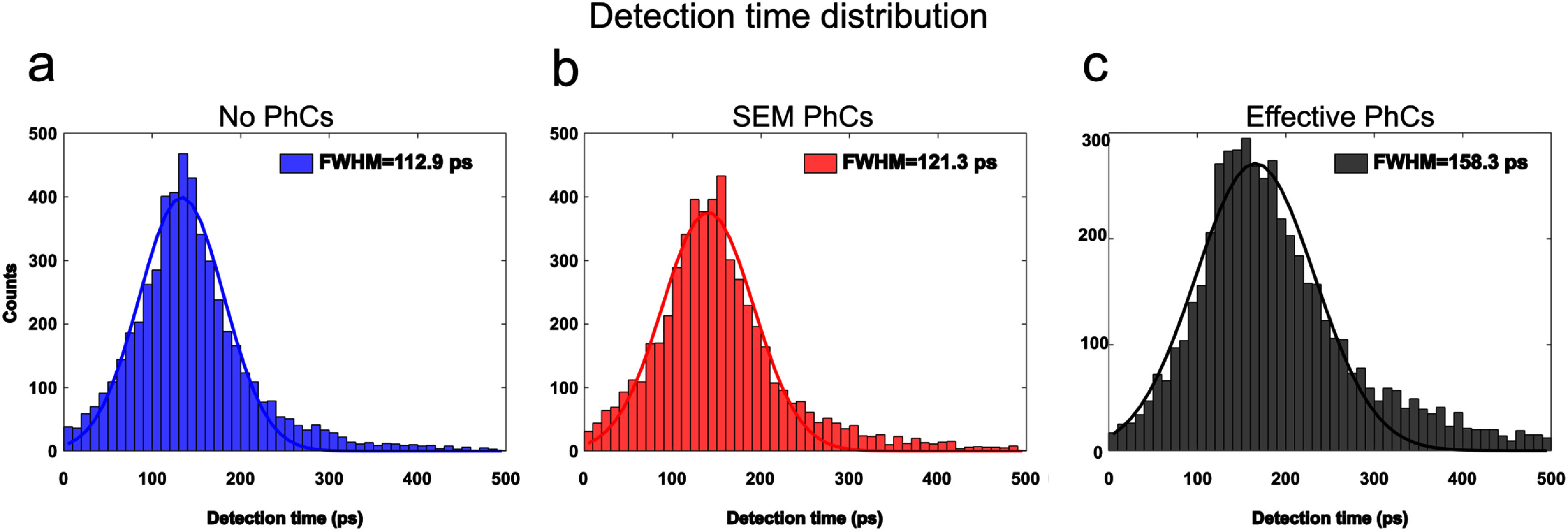
Modeled detection time distribution for different scintillator-photodetector coupling. ‘No PhCs’ is in blue distribution; ‘SEM PhCs’ is in red distribution; and ‘Effective PhCs’ is in gray distribution.

Figure 9(a) shows the simulated optical photons’ transmissivity at different scintillator-photodetectors coupling scenarios. The blue curve represents the polished LYSO coupled with photodetector by glue; the red curve indicates ‘SEM PhCs’ coupling and the black curve shows the ‘Effective PhCs’ coupling. The traditional coupling (No PhCs) has a critical angle around 62°(consistent with Snell’s law), while the two PhCs couplings do not have a clear critical angle. Both PhCs couplings have lower transmissivity at incident angles lower than 60° but higher transmission than the conventional coupling above the critical angle (no PhCs). Figure 9(b) depicts the simulated pulse height spectra for these three scintillator-photodetectors coupling scenarios. The number of collected optical photons was used to represent the pulse amplitude. Each photopeak was fitted with a Gaussian function to estimate the peak positions, FWHM and energy resolution (*E*_r_). The modeled detection time distribution is shown in figure 10. We used a Gaussian model to fit the distributions and estimate their FWHM. The ‘No PhCs’ had FWHM of 112.9 ps; the ‘SEM PhCs’ has 121.3 ps; and the ‘Effective PhCs’ has158.3 ps.

### 3.3. Experimental pulse height spectrum

**Figure 11.**
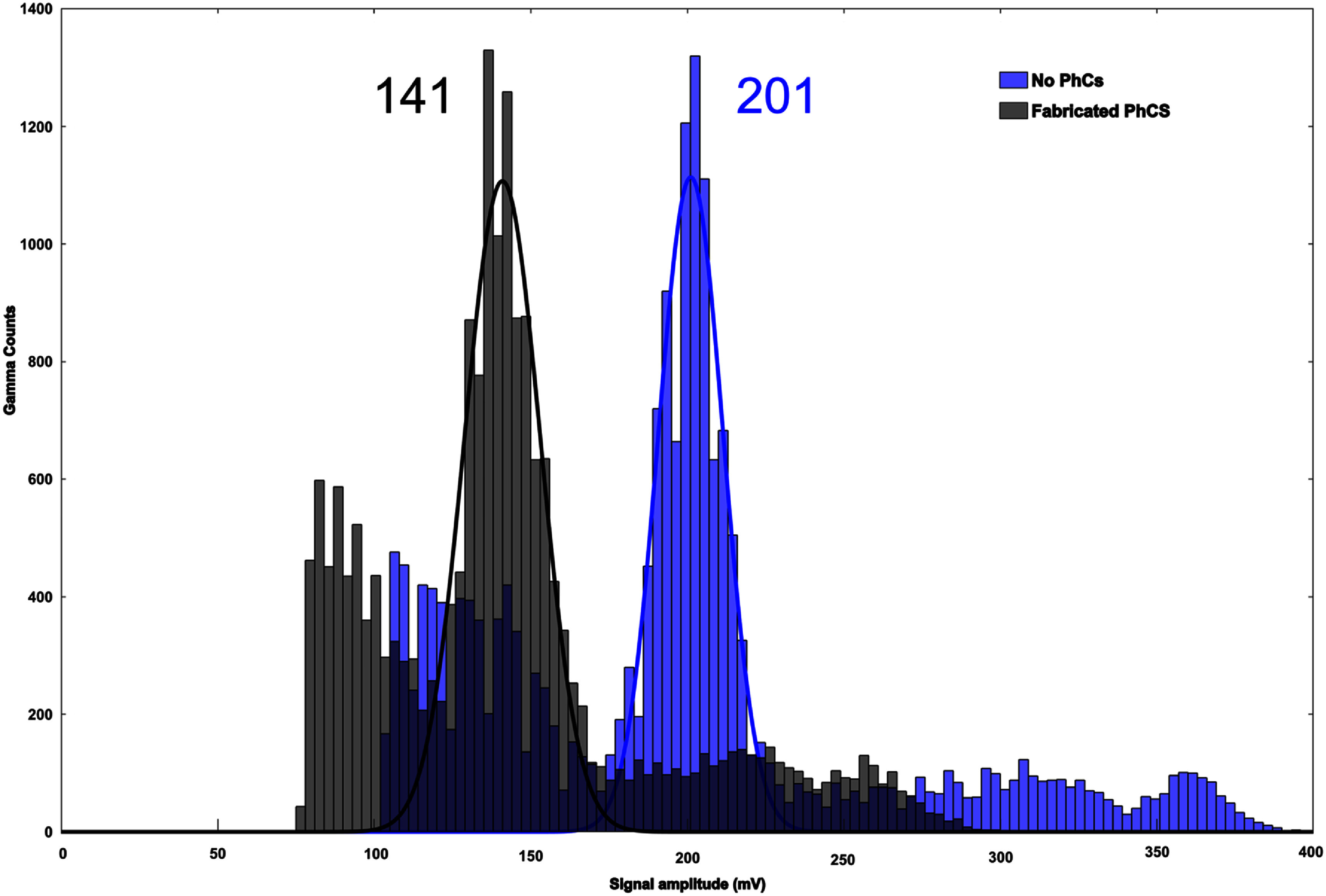
Experimental pulse height spectrum.

Figure 11 shows the experimental pulse height spectrum. The blue distribution represents a ‘pure glue’ coupling case while the black distribution represents the ‘Fabricated PhCs’. We applied a Gaussian fit to the photoelectric events and obtained the photopeak as well as the FWHM. The ‘No PhCs’ photopeak position was 201 mV and the FWHM was 24 mV, while the ‘Fabricated PhCs’ photopeak position was 141 mV with a FWHM of 28 mV. Therefore, the ‘No PhCs’ has an energy resolution of 12% and the ‘Fabricated PhCs’ energy resolution of 20%. Based on the measured photopeak positions, the ‘No PhCs’ coupling can collect more optical photons than the ‘Fabricated PhCs’ (table 1). In simulations, the ‘No PhCs’ collects fewer optical photons than ‘SEM PhCs’ while collecting more than ‘Effective PhCs’, showing that the ‘SEM PhCs’ does not reproduce the experimental trend. These results indicate that the ‘Effective PhCs’ model is closer to the ‘Fabricated PhCs’ than the SEM model. Both simulations and experiments indicate a reduction in optical photon collection, although the magnitude of the reduction differs: the simulations show a 13% reduction from the ‘No PhCs’ to the ‘Effective PhCs’, and the experiments show a great light collection loss of 30% reduction with the ‘Fabricated PhCs’.

**Table 1.** Summary of simulation and experiment results.

	Simulation			Experiment	
	No PhCs	SEM PhCs	Effective PhCs	No PhCs	Fabricated PhCs

Photopeak	2082 counts	2514 counts	1806 counts	201 mV	141 mV
Photopeak change relative to ‘No PhCs’	N/A	21%	−13%	N/A	−30%
Gaussian fitting coefficient	0.96	0.93	0.96	0.91	0.90
FWHM	273 Counts	331 Counts	274 Counts	24 mV	28 mV
*E*_r_	13%	13%	15%	12%	20%
Detection time resolution	112.9 ps	121.3 ps	158.3 ps	98.5 ps	115 ps

**Figure 12.**
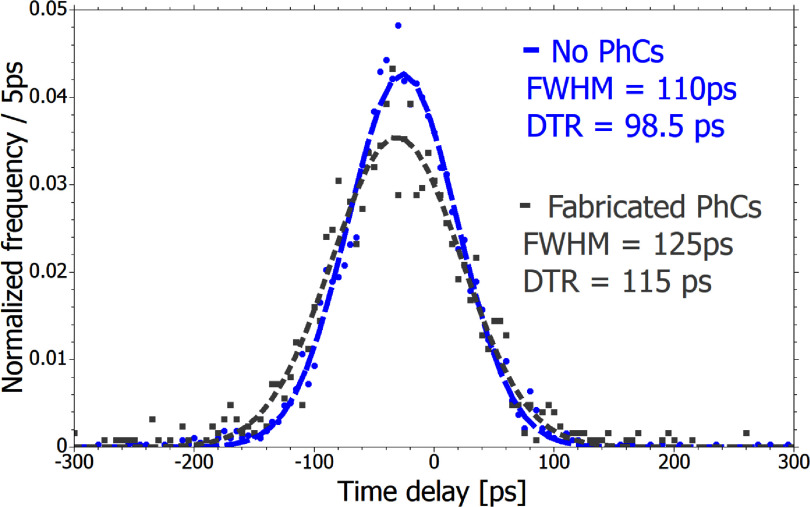
Experimental coincidence timing resolution for both coupling scenarios. Blue markers and curve represent the ‘No PhCs’, and gray markers and curve represent the ‘Fabricated PhCs’. DTR refers to detection timing resolution.

The experimental coincidence timing distribution is shown in figure 12 and table 1 last row. ‘Blue’ and ‘gray’ markers represent ‘No PhCs’ and ‘Fabricated PhCs’ couplings. The reference detector has detection time resolution (DTR) around 49.5 ps, as mentioned before with a CTR (for two identical reference detectors) of 70 ps FWHM. We used a Gaussian model to fit the distributions and estimate their FWHM in figure 12. The ‘No PhCs’ had DTR of 98.5 ps; the ‘Fabricated PhCs’ had DTR of 115 ps. Both simulation method and experimental method showed that ‘Fabricated PhCs’ could degrade timing resolution than ‘No PhCs’.

## 4. Discussion

The discrepancy between the SEM model and experimental results can be explained by the PhCs’ fabrication process: (1) nanoimprinting technology to deposit TiO_2_ unit cells upon LYSO surface with a periodic pattern, (2) annealing, and (3) encapsulation of TiO_2_ unit cells with SiO_2_. Steps 2 and 3 may have distorted and reduced the PhCs’ unit cell, which were already apparent in figure 1(a) but could not be directly measured to adjust the PhCs model. The annealing process could have twisted some nanostructures, giving them a Gaussian shape. The encapsulation process could have reduced the nanostructures’ height, which led us to test the effect of the height on light collection (figure 4(b)). Namely, the longitudinal cross-section of a 3D Gaussian in figure 4 clearly shows the effect of encapsulation: the ‘Effective PhCs’ optimized height and lattice distance needed to be set to 300 nm and 650 nm so that we could reasonably match the modeled far field with the experimental far field. However, if we input ‘SEM PhCs’ structures into Ansys Lumerical to simulate the far field, the modeled far field showed a huge discrepancy with the experimental far field. After determining the optimal ‘Effective PhCs’ geometry, we could compute its transmission curve, shown in black in figure 9(a), by inputting the geometry into Ansys Lumerical FDTD. Both the ‘Effective PhCs’ transmission curve and SEM PhCs’ removed the critical angle but the ‘Effective PhCs’ transmission had a much lower transmissivity drop at smaller incident angles compared to ‘SEM PhCs’ and ‘No PhCs’. Fabrication duration, annealing, and encapsulation each can contribute to the transmissivity degradation at smaller incident angles because these processes distort the PhCs’ unit structure and reduce their height after initial nanoimprinting. The energy resolution with PhCs also became worse than ‘No PhCs’ in both simulations and experiments (table 1). The ‘Effective PhCs’ model predicts a 13% LY_coll_, while experiments showed a 30% reduction. There is some uncertainty on the lattice distance ($ \pm 20$ nm), which may contribute to the LY_coll_ discrepancy. However, building LUT with different unit cells’ geometries is time consuming, which is around 4 months for one PhCs’ unit cell geometry. To more accurately model the scintillator-PhCs detector performance, we could segment the scintillator’s cross-section area into multiple regions and apply different PhCs LUTs to different regions to represent the heterogeneity of the PhCs patterning, especially the rotation of the hexagonal lattice visible in figure 1(a). The light output simulated with the ‘Effective PhCs’ model better matched the experimental light output, indicating that this model may be applied to simulation-guided refinement of PhCs designs and fabrication with fewer defects, and ultimately to performance-driven PET detector development. We only had one LYSO-PhCs sample with a size of 3 × 3 × 20 mm^3^ in this work. In the future, we will fabricate more LYSO-PhCs samples with different geometries and characterize their *E*_r_ and DTR. Some groups have used rigorous coupled-wave analysis (RCWA) to solve the Maxwell equations solutions for PhCs (Knapitsch and Lecoq 2014) (Liu *et al* 2015) (Johnson and Joannopoulos 2002). However, RCWA solved the Maxwell equations in frequency domain while FDTD solved the equations in time domain. RCWA can only solve the ideal PhCs equations, but FDTD can solve the non-periodic structure which is ‘Effective PhCs’.

## 5. Conclusion

The fabricated PhCs deposited on LYSO-Photodetector interface may not always increase the optical photons detection efficiency in PET detectors. This is due to defects in PhCs structure resulting from the fabrication process. These PhCs can downgrade the optical photons efficiency. This undesired effect was not reproduced with our original PhCs LUT model (He *et al* 2025). In this new work, we established a new approach to model non-ideal PhCs, combining experimental characterization and modeling that could be applied to new PhCs samples. The optical photon harvest better matched experimental results. Therefore, our method to model non-ideal PhCs can be used in the future to design and optimize PET detectors based on PhCs, based on a simple experimental characterization of the PhCs crystals with a laser that can be performed by end users. An alternative to refine the SEM PhCs model would require accessing a precise measurement of the final height of the nanostructures to use the exact height to define the unit cell in the FDTD computation during fabrication.

## Data Availability

The data cannot be made publicly available upon publication because no suitable repository exists for hosting data in this field of study. The data that support the findings of this study are available upon reasonable request from the authors.
